# Nivolumab for Metastatic Urothelial Cancer in a Renal Allograft Recipient With Subsequent Graft Rejection and Treatment Complete Remission: A Case Report

**DOI:** 10.3389/fonc.2021.646322

**Published:** 2021-05-26

**Authors:** Didi Chen, Xinyi Wu, Congying Xie

**Affiliations:** Department of Radiation and Medical Oncology, The First Affiliated Hospital of Wenzhou Medical University, Wenzhou, China

**Keywords:** kidney transplantation, urothelial cancer, nivolumab, graft rejection, complete remission

## Abstract

Immune checkpoint inhibitors (ICIs) expanded the therapeutic options for several cancers. However, whether some special groups of patients including those with organ transplantation can receive ICIs remains unclear. In this report we presented an interesting case. A 54-year-old woman underwent kidney transplantation, developed metastasis 7 years after operation of the bladder tumor. Her disease progressed after chemotherapy and radiotherapy. Anti-PD-1 immunotherapy was then considered. After two cycles of nivolumab immunotherapy, the patient’s renal function declined rapidly. Acute allograft rejection was considered. There was no significant decrease in creatinine after glucocorticoid pulse therapy. Third course of nivolumab was given, and regularly hemodialysis was simultaneously conducted. Two weeks later, the patient showed left abdominal pain. CT scan revealed a reduction in tumor burden, while enlarged volume of kidney graft. Immunotherapy stopped. Two months after the third course, CT demonstrated a complete remission to immunotherapy. 23 months after the third course, CT showed that the swelling transplanted kidney was smaller than previous, and no recurrence was observed.

## Introduction

Bladder cancer is a common malignant tumor of urinary system, 90% of which are urothelial cancer (1). During the past several decades, limited progress has been made in the treatment for locally advanced or metastatic bladder cancer. Immune checkpoint inhibitors (ICIs) have revolutionized the field of oncology, becoming a novel strategy for anticancer therapy. Representative ICIs, the programmed death 1 (PD-1) receptor and one of its ligands (PD-L1), have been approved for the treatment of urothelial cancer. Compared with chemotherapy, ICIs have advantages of long duration of response and low rate of adverse events.

However, it remains unclear whether organ transplant patients can receive ICIs. Previous evidence suggests that PD-1 and PD-L1 pathways participate in the process of transplant tolerance and prevention of chronic allograft rejection (2). Antibodies targeting PD-1 or PD-L1 block PD-1 or PD-L1, thus activating anti-tumor immune reaction (3). Previous ICIs clinical trials excluded patients who had undergone organ transplantation. There are few case reports of these patients treated with ICIs.

Here, we describe a case of a renal allograft recipient with metastatic urothelial cancer who achieved complete remission after treatment with PD-1 inhibitor. Although the patient developed acute rejection after anti–PD-1 therapy, resulting in treatment discontinuation and renal failure.

## Case Report

**Figure 1:**
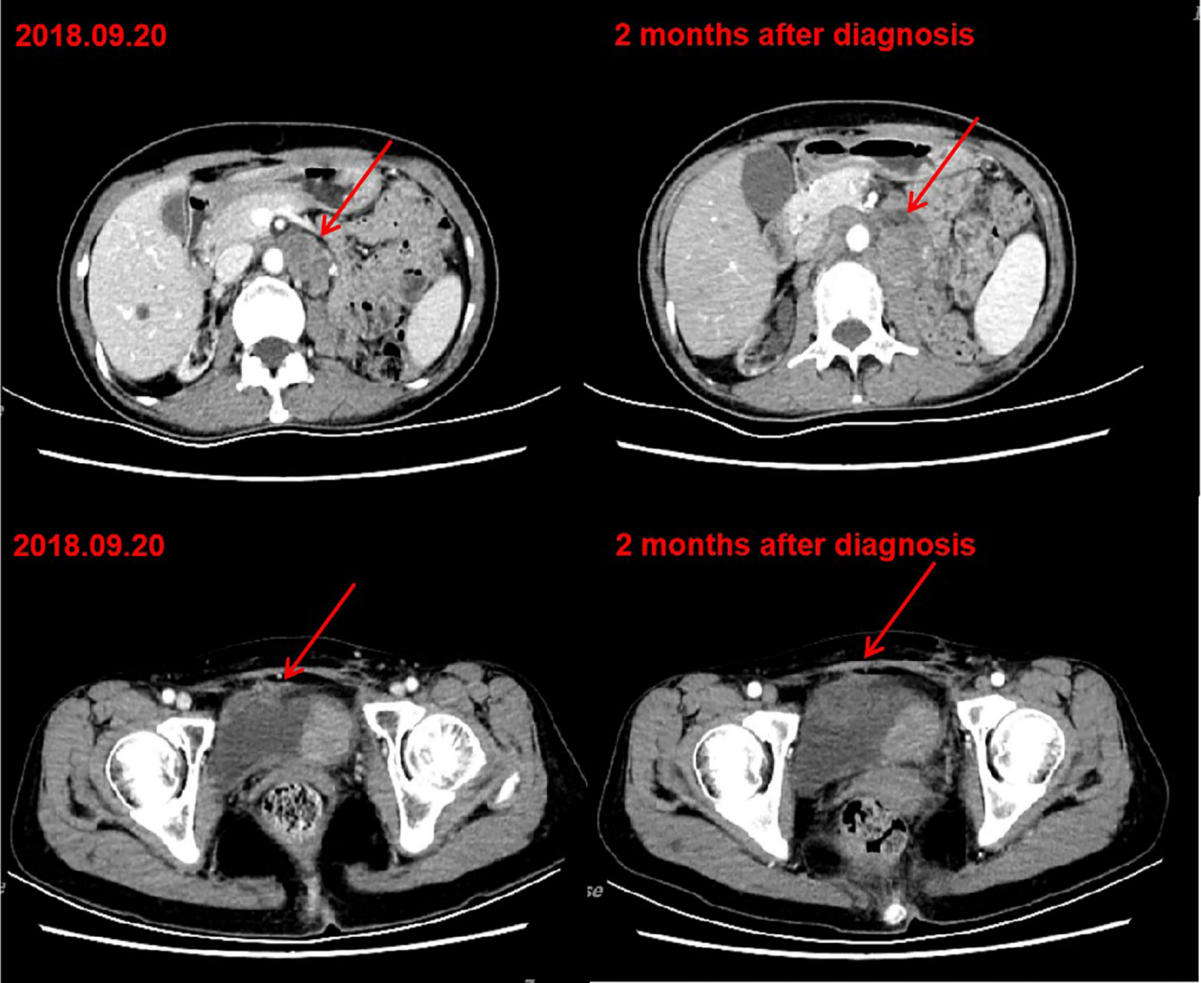
CT after chemotherapy indicated tumor progression. Left: CT of September 20, 2018. Bladder cancer with retroperitoneal and pelvic lymph node metastasis. Right: CT of November 12, 2018. After two cycles of gemcitabine chemotherapy, retroperitoneal and pelvic lesions progressed.

A 54-year-old woman received a deceased donor kidney transplant in 2005 for end-stage renal disease secondary to aristolochic acid nephropathy. Before transplantation, the serum creatinine level of the patient was between 600-750 μmol/L. The human leukocyte antigen (HLA) compatibility of donor to recipient showed a mismatch of four antigens. The patient had been receiving tacrolimus 1.0 mg twice daily and mycophenolate mofetil 500 mg twice daily without any evidence of chronic rejection. The target trough level of tacrolimus was maintained between 3 ng/ml and 4 ng/ml. The transplanted kidney had been functioning well, with baseline serum creatinine level of <80 μmol/L. There was persistent low level proteinuria (4-5mmol/L) and there were no detectable HLA antibodies. In 2011, 6 years post-transplant, the patient was diagnosed with bladder urothelial cancer and underwent subsequent transurethral resection. The surgical pathological staging was 0a. In 2016, the immunosuppression regimen was changed to tacrolimus 1.0 mg twice daily and enteric-coated mycophenolate sodium 180 mg twice daily. In May 2018, the patient was diagnosed with left renal pelvic carcinoma and underwent radical nephrectomy. The result of surgical pathology was stage IIIa renal pelvic urothelial carcinoma. In September 2018, she had a local recurrence in the bladder and developed metastatic disease in the pelvic lymph nodes and retroperitoneal lymph nodes. Considering the nephrotoxicity of cisplatin and her poor candidacy for doublet chemotherapy [Eastern Cooperative Oncology Group (ECOG) performance status of 2 (on a 5-point scale, with higher scores indicating greater disability)], she received gemcitabine chemotherapy at 1000mg/m^2^ on days 1 and 8 every 3 weeks. After two cycles of gemcitabine treatment, the patient’s disease continued to progress (**Figure 1**).

A retroperitoneal lymph node biopsy was performed, which confirmed metastatic carcinoma. Due to edema of her left leg and hematuria, palliative radiotherapy was administered to the retroperitoneal lymph nodes (46 Gray in 23 fractions) and related bladder areas (46 Gray in 23 fractions). Genetic testing, including 448 genes and tumor mutation burden (TMB), was performed in retroperitoneal lymph node biopsy samples. A total of 97 somatic mutations were identified. Among them, only the TP53 gene mutation was previously reported to be sensitive to several cytotoxic drugs. In addition, her TMB was high (66.44 MutS/MB). CT scan performed in February 2019 revealed regression of retroperitoneal lymph nodes and bladder tumor areas receiving radiotherapy, while progression of pelvic lymph nodes and appearance of several new liver lesions.

**Figure 2:**
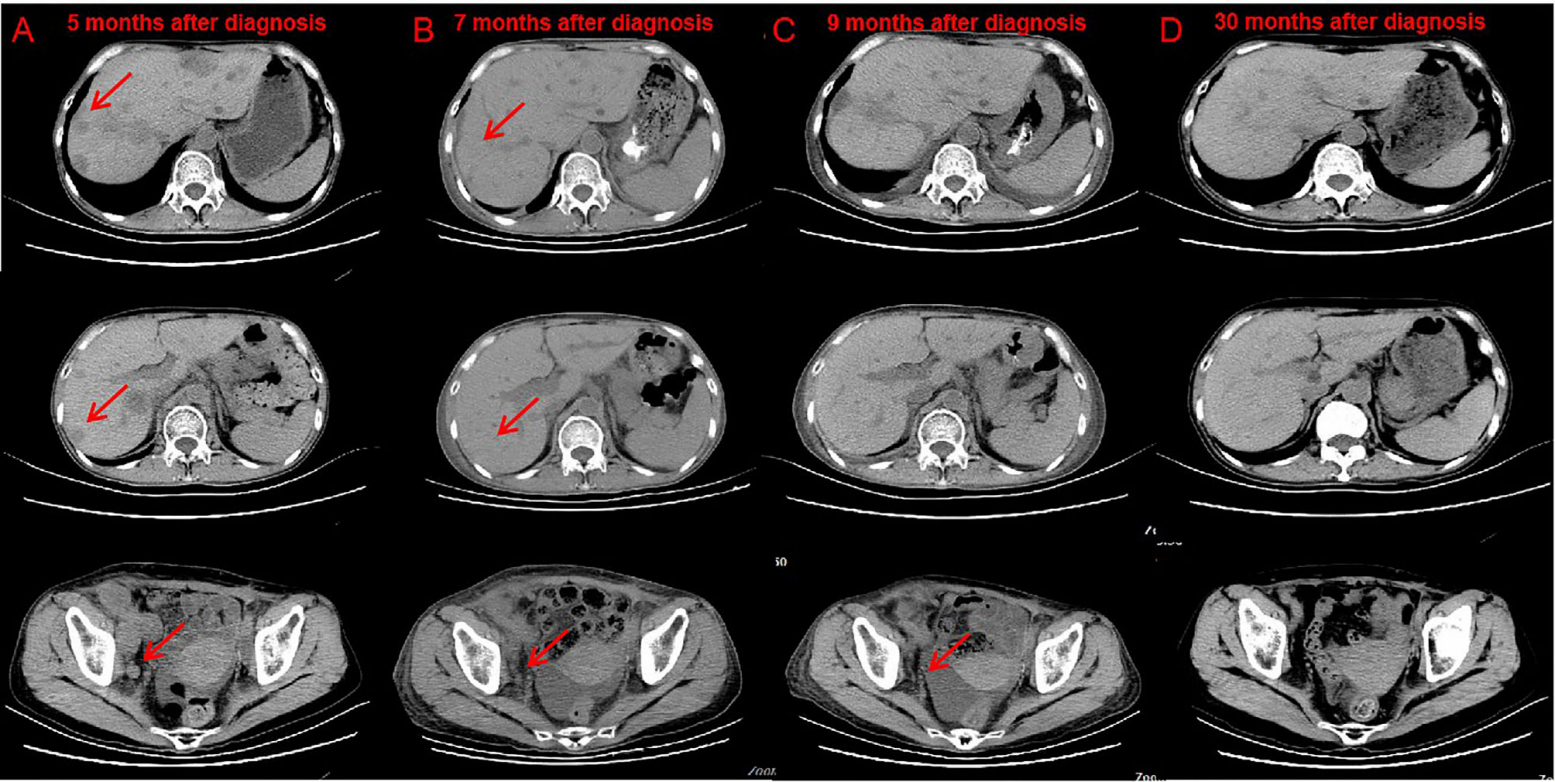
CT after nivolumab indicated tumor response. Compare CT of April 18, 2019 **(B)** with February 20, 2019 **(A)** Bladder cancer with liver, abdominal cavity, retroperitoneal, pelvic, bilateral inguinal lymph node metastasis, part of the liver lesions and lymph nodes shrink. CT of June 3, 2019 **(C)** and March 2, 2021 **(D)** showed no lesions were found in the liver, abdominal cavity, retroperitoneal, pelvic.

**Figure 3:**
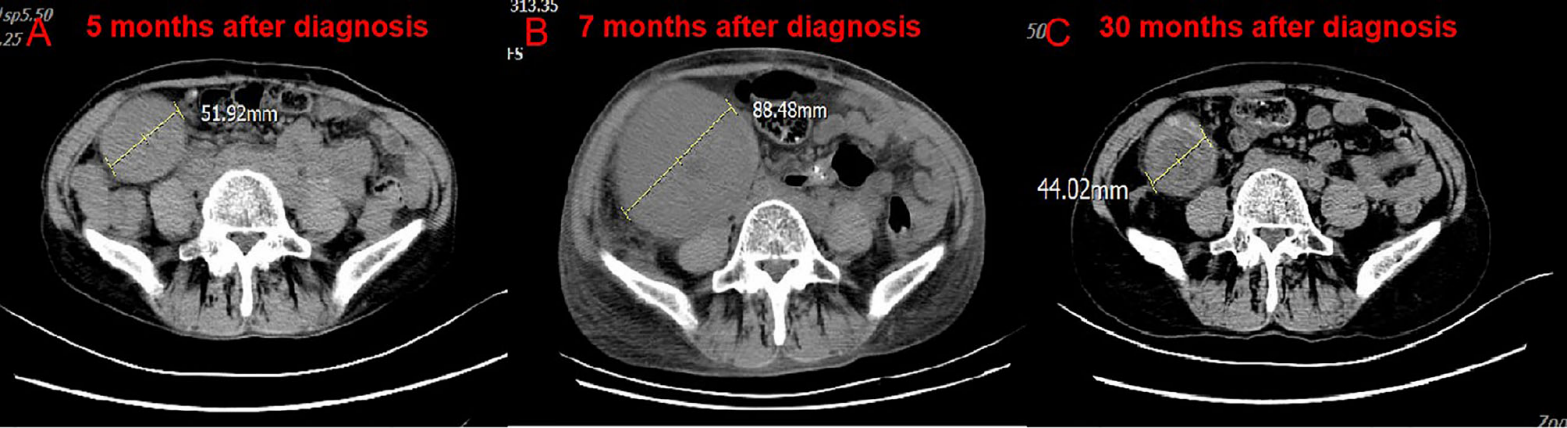
CT scan of the volume of transplanted kidney. Compare CT of April 18, 2019 **(B)** with February 20, 2019. **(A)** The volume of transplanted kidney increased after treatment of nivolumab. Compare CT of March 2, 2021 **(C)** with April 18, 2019. **(B)** The volume of transplanted kidney decreased after nivolumab stopped.

Administration of anti–PD-1 was then considered on the basis of her rapid disease progression and the potential immunotherapy benefits of anti–PD-1 for patients with high TMB. Before starting immunotherapy, the risks of immune-related toxic effects associated with PD-1 inhibitors, including kidney allograft rejection, were explicitly conveyed to the patient. Tacrolimus was discontinued, and enteric-coated mycophenolate sodium was reduced to 180 mg twice daily. Nivolumab was given in 3 mg/kg every 2 weeks. CT performed after the first cycle of nivolumab revealed a reduction in tumor burden, which was assessed as a stable disease. A week after the second dose, the serum creatinine level exceeded 200 μmol/L. Acute rejection was considered. No donor specific antibodies were identified. High-dose intravenous methylprednisolone treatment (300 mg d1, 200 mg d2, 125 mg d3) was initiated, followed by tapering of prednisone once acute allograft rejection was diagnosed. Meanwhile she received mycophenolate sodium 180 mg twice daily and tacrolimus 0.5 mg twice daily. The target trough level of tacrolimus was controlled between 2 ng/ml and 3 ng/ml. Serum creatinine level slightly decreased at first, then increased markedly (**Figure S1**). After discussion with the patient herself and her family members, a third course of nivolumab was administered, and regularly hemodialysis was simultaneously conducted. During hemodialysis, she developed hypertension, the blood pressure was up 200/120mmHg. Two weeks after the third course of nivolumab, the patient developed left abdominal pain, and physical examination revealed palpable left kidney. CT scan demonstrated a partial response to immunotherapy (**Figures 2A, B**), and an increase in the kidney graft volume (**Figures 3A, B**). As the patient declined a renal biopsy, acute rejection could not be confirmed. The patient’s performance status declined. Considering that she couldn’t tolerate immunotherapy, anti-tumor treatment discontinued. Her hypertension was slowly brought under control with medication. Two months after the third course (June, 2019), CT revealed a complete response to immunotherapy (**Figure 2C**) and further swelling of the transplanted kidney. Four months after the third course (August, 2019), CT scan indicated a persistent complete response, and the swelling transplanted kidney was smaller than previous. Mycophenolate sodium was discontinued two months later, immunosuppression therapy continued with tacrolimus 0.5 mg twice daily. Regular CT scan was carried out every three months. 23 months after the third course (March, 2021), CT confirmed a further decrease in swelling of the transplanted kidney and restoration of the kidney to its former volume (**Figure 3C**). No recurrence was observed (**Figure 2D**). She remains dependent on hemodialysis.

## Discussion

Immune checkpoint inhibitors (ICIs) have expanded the therapeutic options for several types of cancers. Patients with organ transplantation were excluded from previous ICIs clinical trials. Case reports of patients treated with ICIs are also limited. Kumar et al. reported that the disease control rate was 45% in 64 transplant patients receiving immunotherapy. Among them, twenty six (41%) developed graft rejection after receiving a median of 2 cycles (1-11 cycles) of PD-1 inhibitors. In cases with rejection, 8 (29%) grafts could be saved, and the rest suffered permanent graft failure (4). Overall, the risk rate of rejection and graft failure were relatively high. Therefore, for transplantation patients, it is highly necessary to carefully weigh the advantages and disadvantages of immune checkpoint inhibitors.

Previous studies focusing on ICI use in transplantation patients mainly included those with melanoma, who responded well to ICIs. Only one case reported tumor regression after combination therapy with anti-PD-1 immunotherapy in a renal transplant recipient with urothelial cancer (5). In our case, the patient’s metastatic urothelial cancer achieved a complete response after PD-1 inhibitor treatment alone. A single-center cohort study confirmed that renal transplant recipients with aristolochic acid nephropathy are at higher risk of developing urothelial carcinoma (6). The median overall survival of patients with urothelial carcinoma after first-line platinum chemotherapy failure is only 7 months (7). Limited treatment options are available for these patients. Recently, PD-1 inhibitors have been reported to improve the objective response rate and overall survival of advanced second-line urothelial carcinoma (8–11). CheckMate 275 reported that 2% of all patients had a complete response and 17% of all patients had a partial response to nivolumab out of 270 patients with metastatic or surgically unresectable urothelial carcinoma after first-line treatment enrolled (7). In KEYNOTE-045, pembrolizumab significantly improved the median overall survival (10.3 months) compared with chemotherapy (7.4 months) in patients with metastatic urothelial carcinoma. The median duration of response was not reached in the pembrolizumab group (range, 1.6+ to 15.6+ months) (9). Among a broad variety of cancer types, higher somatic TMB is associated with better overall survival in patients receiving ICIs (12). Therefore, PD-1 inhibitor was administered to the patient after the patient and her family were informed about the potential risks of graft failure.

Although the patient developed acute rejection after anti-PD-1 therapy, resulting in treatment discontinuation and renal failure. So far, the patient showed a complete response for 23 months. Tumor cells are known for their ability to escape from T-cell-mediated immunosurveillance and inhibit the effector response by upregulating inhibitory checkpoint molecules, such as the programmed death ligands PD-L1 and PD-L2, which interact with PD-1 on T cells to suppress their activation (13). The PD-1/PD-L1 pathway is also involved in the maintenance of graft tolerance (14). Blockade of PD-1 increases the activation of T cells not specifically against malignant cells, but also other cells expressing foreign antigens such as kidney allograft donor antigens, leading to graft rejection (15). A retrospective cohort study including 69 renal transplant patients reported that the objective response rate to ICI therapy was 28.9%. Among them, 42% developed acute graft rejection, 65.5% of whom did not recover and required dialysis (16). Biopsy confirmed that the most graft failures were caused by acute cellular rejection. PD-1–positive T-cell infiltration in renal tissue after treatment with PD-1 inhibitors has been found in excised kidney from transplanted patients (17). However, not all transplant patients developed graft rejection after receiving PD-1 inhibitors. Murakami et al. reported that mTOR inhibitor use and triple-agent immunosuppression was associated with a lower risk of rejection (16). The mTOR pathway regulates many processes such as cell survival, metabolism and growth. Treatment with mTOR inhibitors has showed efficiency in several cancer types (18). The effect of mTOR inhibitor may be understood from the viewpoint that mTOR signaling pathway plays a role not only in immunosuppression after solid organ transplantations, but also in tumorigenesis and progression. The incidence of acute graft rejection was lower in renal transplant patients with higher number of immunosuppressants at the time of ICI initiation. Moreover, ICIs may still provide a reasonable tumor response (16). Thus, it is uncertain whether the continuation of immunosuppressants reduces the anti-tumor response of ICIs and should be further investigated. Other mechanisms that induce graft rejection in transplant patients after receiving ICIs remain unclear. There is no evidence suggesting that tumor response to ICI was different between patients who did and did not develop acute graft rejection. Therefore, finding optimal treatment regimens to enhance the tumor-specific T-cell response and decrease T-cell-mediated alloreactivity after treatment with ICI in transplant recipients with tumors is worth exploring.

In conclusion, in this case, PD-1 inhibitor has been proved to be a lifesaving measure. How to balance the risks and benefits of immunotherapy in patients undergoing transplantation is a challenge. In the case of potential unacceptable graft rejection or failure (e.g. heart, lung, liver transplantation), other treatments should be given in priority. As for renal transplant recipients, hemodialysis can be used to substitute the function of kidney. It is worth further exploring whether immunotherapy can be more actively used as an anti-tumor therapy in these patients.

## Data Availability Statement

The original contributions presented in the study are included in the article/**Supplementary Material**. Further inquiries can be directed to the corresponding author.

## Ethics Statement

Written informed consent was obtained from the individual(s) for the publication of any potentially identifiable images or data included in this article.

## Author Contributions

DC wrote the manuscript. XW analyzed the medical record. Manuscript editing was done by CX. All authors contributed to the article and approved the submitted version.

## Funding

This work was supported by the grants from the Key Research and Development project of Zhejiang Province (grant number 2020C03028) and Science and technology cooperation project of Wenzhou science and technology bureau (grant number H20180003).

## Conflict of Interest

The authors declare that the research was conducted in the absence of any commercial or financial relationships that could be construed as a potential conflict of interest.

## Supplementary Material

Supplementary Figure 1 Rise in creatinine in relation to nivolumab.

Supplementary Figure 2 Timeline.
