# Supplementary figures and images for: Nivolumab for Metastatic Urothelial Cancer in a Renal Allograft Recipient With Subsequent Graft Rejection and Treatment Complete Remission: A Case Report
Source: Front Oncol. 2021 May 26;11:646322. doi: 10.3389/fonc.2021.646322 (PMC8187800; doi:10.3389/fonc.2021.646322)

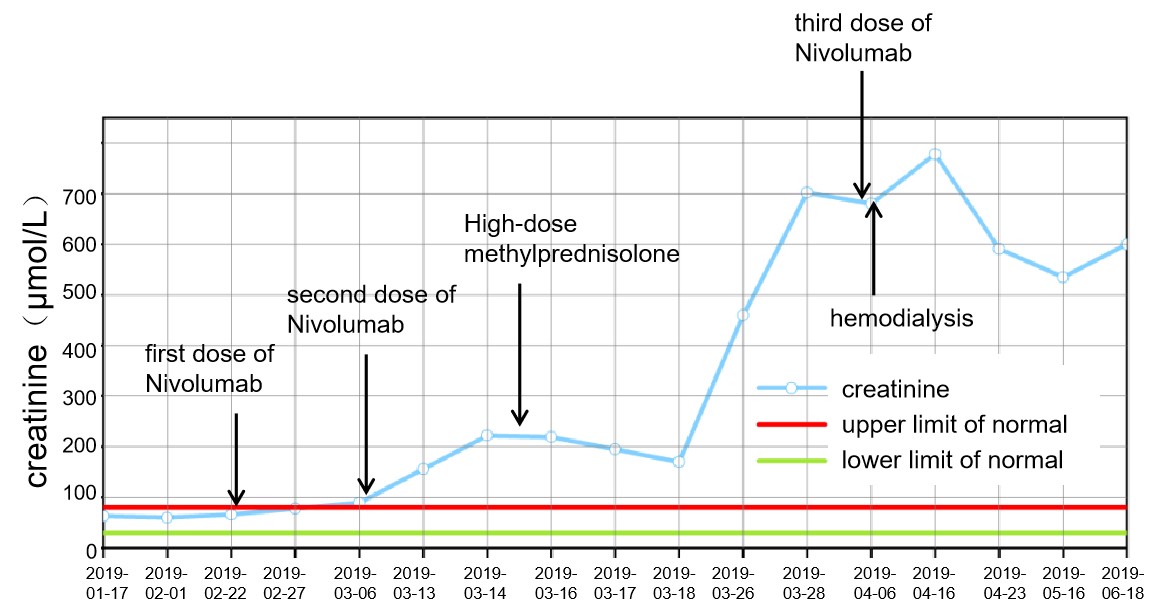

Supplement: Supplementary Figure 1 — Rise in creatinine in relation to nivolumab. [file Image_1.jpeg]

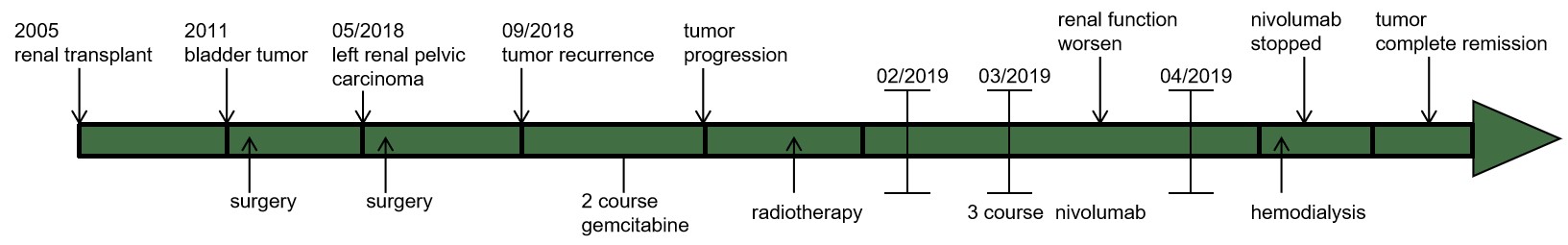

Supplement: Supplementary Figure 2 — Timeline. [file Image_2.jpeg]
